# Diagnosing asthma in general practice with portable exhaled nitric oxide measurement – results of a prospective diagnostic study

**DOI:** 10.1186/1465-9921-10-15

**Published:** 2009-03-03

**Authors:** Antonius Schneider, Lisa Tilemann, Tjard Schermer, Lena Gindner, Gunter Laux, Joachim Szecsenyi, Franz Joachim Meyer

**Affiliations:** 1 Department of General Practice and Health Services Research, University Hospital, University of Heidelberg, Heidelberg, Germany; 2 Department of Primary Care Medicine, Radboud University Nijmegen Medical Centre, Nijmegen, the Netherlands; 3 Department of Cardiology, Pulmonology and Angiology, Medical Centre, University of Heidelberg, Heidelberg, Germany

## Abstract

**Background:**

To evaluate the sensitivity, specificity and predictive values of fractional exhaled nitric oxide (FENO) for the diagnosis of asthma in general practice.

**Methods:**

Prospective diagnostic study with 160 patients attending 10 general practices for the first time with complaints suspicious of obstructive airway disease (OAD). Patients were referred to a lung function laboratory for diagnostic investigation. The index test was FENO measured with a portable FENO analyser based on electrochemical sensor. The reference standard was the Tiffeneau ratio (FEV_1_/VC) as received by spirometric manoeuvre and/or results of bronchial provocation. Bronchial provocation with methacholine was performed to determine bronchial hyper-responsiveness (BHR) in the event of inconclusive spirometric results.

**Results:**

88 (55%) were female; their average age was 43.9 years. 75 (46.9%) patients had asthma, 25 (15.6%) had COPD, 8 (5.0%) had an overlap of COPD and asthma, and 52 (32.5%) had no OAD. At a cut-off level of 46 parts per billion (ppb) (n = 30; 18.8%), sensitivity was 32% (95%CI 23–43%), specificity 93% (95%CI 85–97%), positive predictive value (PPV) 80% (95%CI 63–91%), negative predictive value (NPV) 61% (95%CI 52–69%) when compared with a 20% fall in FEV_1 _from the baseline value (PC_20_) after inhaling methacholine concentration ≤ 16 mg/ml. At 76 ppb (n = 11; 6.9%) specificity was 100% (95%CI 96–100%) and PPV was 100% (95%CI 72–100). At a cut-off level of 12 ppb (n = 34; 21.3%), sensitivity was 90% (95%CI 79–95%), specificity 25% (95%CI 17–34%), PPV 40% (95%CI 32–50), NPV 81% (95%CI 64–91%) when compared with a 20% fall of FEV_1 _after inhaling methacholine concentration ≤ 4 mg/ml. Three patients with unsuspicious spirometric results have to be tested with FENO to save one bronchial provocation test.

**Conclusion:**

Asthma could be ruled in with FENO > 46 ppb. Mild and moderate to severe asthma could be ruled out with FENO ≤ 12 ppb. FENO measurement with an electrochemical sensor might be reasonable with respect to the time consuming procedure of bronchial provocation, which carries also some risk of severe bronchospasm. Further research is necessary to evaluate the effectiveness of this dual diagnostic strategy. The number needed to diagnose might be improved when the diagnostic precision could be enhanced by future technical developments.

## Introduction

Asthma is a common chronic disease with a high prevalence of approx. 5% in industrialized nations. It is characterised by an inflammation process which induces bronchial hyper-responsiveness and usually reversible airway obstruction [1]. General practitioners have a key role in detecting the disease, as in most times patients initially come to them with complaints which are suspicious of asthma. Spirometric investigation is seen as being a gold standard for diagnosing obstructive airway disease (OAD) [2]. Efficacy of spirometry in diagnosing severe asthma has already been demonstrated [3]. In mild asthma in particular, an airway obstruction is often not present, thus leading to diagnostic uncertainty. Serial peak-flow measurement or bronchial provocation is recommended in international guidelines for these cases [2]. However, the low diagnostic value of peak-flow variability has already been demonstrated [4,5]; and bronchial provocation thus remains as a gold standard for determining bronchial hyper-responsiveness [6]. Therefore in Germany, patients with complaints suspicious for OAD are referred to a pneumologist for bronchial provocation, if they have inconclusive spirometric results in general practice. Bronchial provocation is indeed time consuming, costly, only available in specialised centres, and carries a small risk of inducing severe bronchospasm [7].

A promising non-invasive and easily available method for diagnosing asthma seems to be the measurement of fractional exhaled nitric oxide (FENO), and increased FENO concentrations have been found in asthmatic patients including those with mild disease [8,9]. Increased FENO is also found in other inflammatory disorders including sinus disease [10] and viral upper respiratory tract infection [11], but not in patients suffering from chronic obstructive pulmonary disease (COPD) [12]; and a high correlation between FENO and conventional tests for diagnosing asthma was demonstrated [13]. However, test characteristics derived from hospital studies are of limited value in primary care due to the lower incidence and smaller extent of the particular diseases found there [14]. Dupont et al. attempted to evaluate the diagnostic accuracy of FENO for primary care patients [15]. They found a specificity of 90% and a positive predictive value of > 90% of FENO in patients referred from general practice to an asthma outpatient clinic. Berkman et al. found a sensitivity of 82.5% and specificity of 88.9% in primary care patients [16]. In both trials a chemoluminescence analyser was used. However, the use of this tool has until now been confined to secondary care because of the expense and physical size of the equipments required to undertake the measurement. A portable hand-held device with an electrochemical sensor (NioxMino^®^) was introduced recently, which was suggested to have a clinically acceptable agreement with a chemoluminescence device (Niox^®^) [17]. The NioxMino^® ^was also evaluated by Menzies et al. [18], who found likewise a high correlation between NioxMino^® ^and Niox^®^. Indeed this instrument was tested in patients with previously established diagnoses which could lead to distorted estimation [19], and the predictive values in relation to different cut-off values were not determined.

To close this gap, the aim of this study was to investigate the sensitivity, specificity and predictive values including the determination of an ideal cut-off value of a portable FENO analyser (NioxMino^®^) for diagnosing asthma in primary care patients. Besides, we wanted to assess the impact of FENO measurement to reduce referrals from primary care for bronchial provocation testing.

## Methods

### Design and Sample

This prospective diagnostic study was performed between February 2006 and June 2007 with fourteen general practitioners (GPs) working in ten German general practices. 160 patients presenting to their GP for the first time with complaints suggestive of obstructive airway disease (OAD) were consecutively included. Inclusion criteria were the presentation of symptoms such as dyspnoea, coughing or expectoration for more than two months, thus leading to clinical suspicion of obstructive or restrictive airway disease as most important differential diagnoses ('indicated population'). GPs were advised to exclude patients with respiratory tract infections preceding the evaluation by 6 weeks. The medical history was recorded using a structured questionnaire (Table 1). The atopic status and rhinitis severity were not evaluated in detail due to the primary care setting of the study. Spirometry was performed in general practice for initial estimation of airway obstruction. Airway obstruction was diagnosed when FEV_1_/VC ≤ 0.70 and/or FEV_1 _< 80% [2]. Lung function reference values corrected for sex, age, and height were used [20]. After initial estimation by their GP patients were sent to the lung function laboratory of the University Medical Hospital. If immediate treatment was necessary due to severe airway obstruction, it was initiated by the GP. Patients were instructed not to use any bronchodilator or inhaled steroid and stop smoking and drinking coffee twelve hours before visiting the lung function laboratory.

**Table 1:** Characteristics of the study population. Values indicate the number (proportion) or mean (SD); OAD = Obstructive Airway Disease; COPD = Chronic Obstructive Airway Disease (n = 160)

	**Asthma** **n (%)**	**COPD** **n (%)**	**Overlap** **n (%)**	**No OAD** **n (%)**
n	75 (46.9)	25 (15.6)	8 (5.0)	52 (32.5)
Female	44 (58.7)	15 (60.0)	4 (50.0)	22 (42.3)
FENO (mean in parts per billion [sd])	42.6 [47.9]	16.2 [11.1]	20.4 [18.6]	24.7 [16.0]
Age (mean in years [sd])	38.7 [15.1]	55.7 [11.9]	63.5 [10.5]	42.8 [15.8]
FEV_1 _(mean of absolute values in litre [sd])	3.43 [0.76]	2.12 [0.73]	1.93 [0.55]	3.52 [0.92]
FEV_1 _(mean of % of predicted [sd])	100 [12.2]	67.8 [18.5]	68.8 [18.4]	107.4 [12.8]
FEV_1_/VC (mean of % [sd])	78.45 [7.02]	59.7 [8.4]	58.2 [7.6]	82.1 [5.8]
Do you ever suffer from shortness of breath? (yes)	48 (64.0)	20 (80.0)	6 (75.0)	27 (51.9)
Do you ever suffered from wheezing in your chest? (yes)	39 (52.0)	15 (60.0)	3 (37.5)	19 (36.5)
Do you often suffer from a cough? (yes)	32 (42.7)	15 (60.0)	4 (50.0)	40 (76.9)
Do you often suffer from expectoration? (yes)	19 (25.3)	10 (40.0)	3 (37.5)	19 (36.5)
Have you ever woken up with a feeling of tightness in your chest? (yes)	19 (25.3)	4 (16.0)	3 (37.5)	8 (15.4)
Have you ever been woken up by an attack of shortness of breath? (yes)	21 (28.0)	3 (12.0)	2 (25.0)	9 (17.3)
Have you ever had an asthma attack? (yes)	7 (9.3)	1 (4.0)	0 (0)	1 (1.9)
Do you suffer from any nasal allergies? (yes)	40 (53.3)	7 (28.0)	1 (12.5)	23 (44.2)
Do you smoke or did you previously smoke? (yes)	30 (40.0)	24 (96.0)	8 (100)	24 (46.2)
How much do/did you smoke? (mean in pack year ([sd])	6.7 [13.3]	35.6 [20.6]	26.5 [17.4]	5.0 [11.1]

Patients with previously established diagnosis of OAD were excluded. Other exclusion criteria related to well known contra-indications for bronchodilator reversibility testing or bronchial provocation, namely untreated hyperthyreosis, unstable coronary artery disease, and cardiac arrhythmia. Pregnancy also led to exclusion. The study was approved by the Medical Ethics Committee of the University of Heidelberg. Patients gave written informed consent.

Based on the pilot study [21], we estimated the pre-test probability of asthma as 45%. In previous trials with a chemoluminescence analyser, sensitivity varied from 82.5% [16] to 69% [15], and specificity from 88 [16] to 80% or respectively 90% [15] (depends on the choice of cut-off point). We conservatively estimated a sensitivity of 69% and a specificity of 80%. Power calculation based on these estimations showed that we had to include at least 149 patients to determine PPV with a 95%CI of ± 10% [22].

### Index Test: FENO – Measurement

All patients underwent the measurement of FENO using the NioxMino^® ^analyzer at a mouth flow rate of 50 mL/s over ten seconds and a pressure of 10 cm H_2_O as per guideline recommendation [23]. A feedback signal of exhalation pressure and exhalation flow was used to control the low flow rate. This procedure was performed at the lung function laboratory of the University Medical Hospital before investigation with bodyplethysmography and bronchial provocation, as forced inspiratory and expiratory manoeuvres could lead to distorted FENO results. The manufacturer Aerocrine^® ^recommended an elevated level at FENO > 20 ppb (as intermediate level) and a level of FENO > 35 ppb as a clear indication for an eosinophilic inflammation in adult patients which is supported by several studies [13,18,24,25].

### Reference Test: Bodyplethysmography and Bronchial Provocation

**Figure 1:**
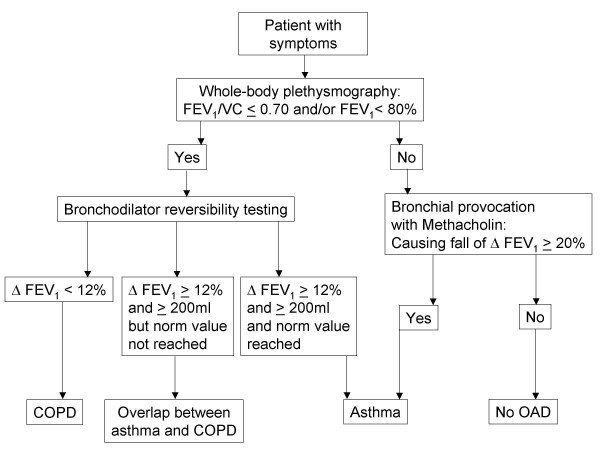
**Diagnostic decision making with the reference standard (COPD = Chronic Obstructive Pulmonary Disease, OAD = obstructive airway disease)**.

The respiratory manoeuvres were performed according to standard protocols [26]. Lung function reference values corrected for sex, age, and height were used [20]. Patients with FEV_1 _< 80% of predicted received a bronchodilation test with an additional performance of whole body plethysmography (WBP) 20 minutes after inhaling salbutamol. An obstructive airway disease was diagnosed if FEV_1_/VC ≤ 0.70. The obstruction was classified as COPD, if the bronchodilation response ΔFEV_1 _after salbutamol was < 12% as compared to baseline and below 200 ml [2] (Figure 1). The obstruction was classified as asthma when ΔFEV_1_was ≥ 12% as compared to baseline and at least 200 ml and lung volumes returned to predicted normal range. An incomplete bronchodilator response was stated if the bronchodilation response was ≥ 12% as compared to baseline and at least 200 ml and lung volumes remained below predicted. We labeled this group 'overlap' as it shows spirometric properties of both, asthma and COPD [27-29]. If there was no bronchial obstruction, bronchial provocation was performed to determine bronchial hyper-responsiveness (BHR). Bronchial provocation is considered to be the best method for diagnosing asthma [6], although there is conflicting evidence [30] probably arising from variations in the populations studied, as the diagnostic value increases with pre-test probability of the disease [31]. Professional lung function technicians measured bronchial hyper-responsiveness to methacholine according to the ATS guidelines [7]. An "asthma" diagnosis was made when there was a 20% fall in FEV_1 _from the baseline value (PC_20_) after inhaling methacholine stepwise until the maximum concentration (16 mg/mL) [7]. The pneumologist was blinded to the FENO results and made the diagnostic decision only on basis of medical history, physical examination, bodyplethysmography and bronchial provocation results.

### Data Analysis

**Figure 2:**
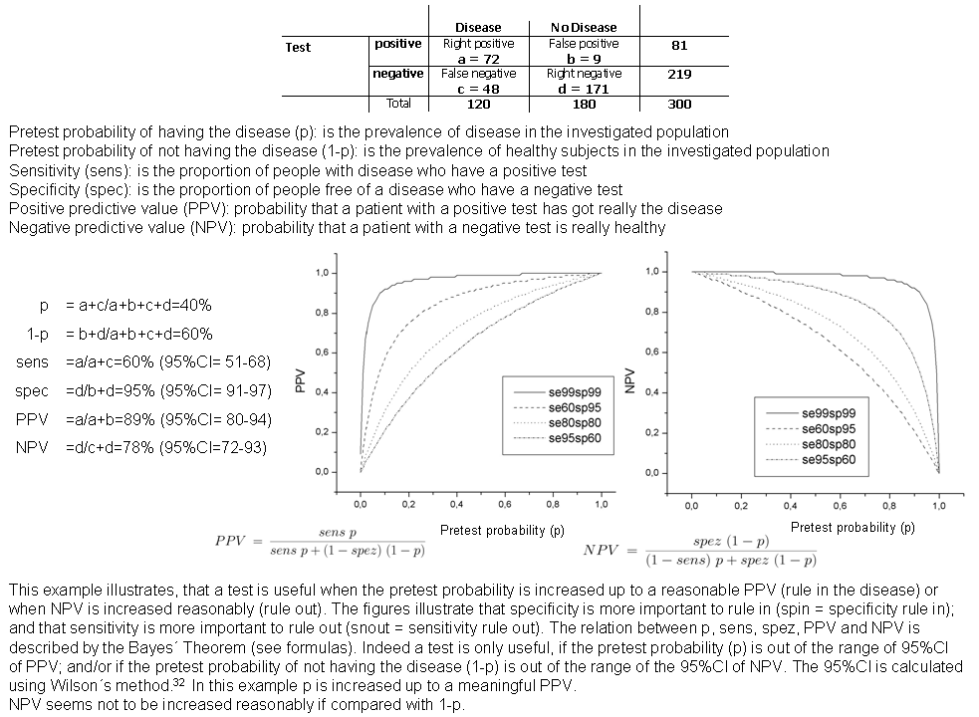
**Calculation example for the relation between pretest probability, sensitivity, specificity, PPV and NPV**.

Baseline data is presented descriptively. Two-by-two contingency tables of FENO values vs. asthma diagnoses (yes or no) were prepared using different levels of FENO as cut-off point. Sensitivity, specificity and predictive values were calculated for each cut-off point. A receiver operating characteristic (ROC) curve was plotted, which allowed a graphical representation of sensitivity and specificity. The cut-off points were analysed with respect to different predictive values. One method of identification is through the highest sum of sensitivity and specificity. Another opportunity is choosing at the highest PPV when NPV was acceptable (or vice versa) at the same cut-off point. Both methods were used. The data was analysed with SPSS 15.0 for Windows. 95% confidence intervals were calculated using Wilson's method [32] with the statistical package CIA (Confidence Interval Analysis) [33]. Positive likelihood ratios (LR+) were calculated to receive the ratio of abnormal finding in ill and healthy subjects. Negative likelihood ratios (LR-) were calculated for the ratio of normal findings in ill and healthy subjects. 95% confidence intervals were derived from the log method [22]. An explanation of how to interpret PPV and NPV is given in figure 2.

The differentiation between asthma, COPD and overlap is a complex problem and sometimes requires repeated measurements after trials of medication. In particular, a negative or incomplete bronchodilation test might be due to a fixed airway obstruction in asthma and not due to COPD. As long term follow up was not possible for organisational reasons, we performed sensitivity analyses with ROC analyses when currently non-smoking patients with less than a five pack year history of nicotine use (initially labelled as COPD or overlap) were classified as asthma patients. Additional sensitivity analyses to control for confounders were performed with exclusion of actually smoking patients [34] and patients using inhaled steroids [35]. Subanalysis was performed for patients with allergic rhinitis in medical history [36].

In particular the diagnostic decision making based on methacholine challenge testing is difficult as there is no full agreement about the cut-off value to be used [30]. The ATS guidelines suggest a cut-off at 16 mg/mL [7], which was also used by Kostikas et al. to evaluate FENO in young adults during pollen season [37]. Berkman determined a cut-off of 3 mg/mL [16], and 8 mg/mL is also commonly used [4,6,15]. Due to this lack of agreement, we calculated cut-off values of FENO with respect to different concentrations of methacholine during bronchial provocation testing, categorised into borderline BHR (4 mg/mL < methacholine concentration ≤ 16 mg/mL), mild BHR (1 mg/mL < methacholine concentration ≤ 4 mg/mL), and moderate to severe BHR (methacholine concentration < 1 mg/mL) following the ATS guideline [7].

## Results

### Study Population

A total of 160 patients participated in the study (88 [55%] female). The recruitment rate was 76%. Patients mostly complained of dyspnea (63.1%), coughing (56.9%), wheezing (45.5%) and allergic rhinitis (44.4%) (Table 1). Seven (4.4%) patients received inhaled steroids (200 to 400 μg budesonide per day) from their GP. Two patients received only short acting β-agonists. Four received long acting β-agonists in addition to inhaled steroids. The duration of treatment was not longer than one or tow weeks as the patients were referred to the lung function laboratory at once. Patients were advised by their GP not to use inhaled medication for twelve hours before lung function testing. According to the results of WBP and bronchial provocation, 75 (46.9%) patients had asthma, 25 (15.6%) had COPD, 8 (5.0%) had an incomplete bronchodilator response (overlap), and 52 (32.5%) patients had no OAD. 36% showed signs of airway obstruction in spirometry in general practice, and 31% showed airway obstruction in the lung function laboratory. Most patients suffered from mild to moderate asthma or COPD, respectively. FEV_1 _and VC values are given in table 1. Three patients had FEV_1 _< 80% and FEV_1_/VC > 0.70; two had asthma (non-smokers), one had COPD (smoker). Patients with asthma had the highest average of FENO levels. Patients with COPD and with an overlap of COPD and asthma were significantly older and had more pack years of smoking than asthma patients (p < 0.001 for each difference; t-Test). At the lung function laboratory the diagnosis of asthma was made in 61 cases with bronchial provocation. According to the ATS guideline categories [7], 17 patients had borderline BHR, 29 had mild BHR and 15 had moderate to severe BHR. Only 14 patients were identified solely on the basis of bronchodilator reversibility testing.

### Estimates of the Diagnostic Accuracy of FENO

**Figure 3:**
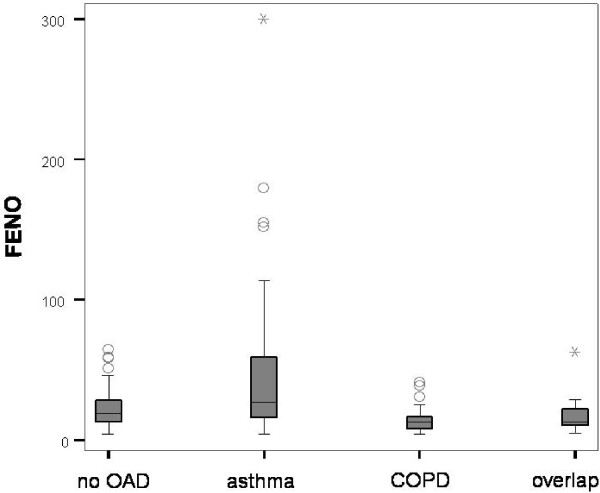
**Box-Plot of FENO measures in relation to the diagnoses of the reference standard (body plethysmography and bronchial provocation)**. (Black circle) mild outlier between 1.5^th ^and 3^rd ^interquartile range. (Black asterisk) extreme outlier more than 3^rd ^interquartile range.

**Figure 4:**
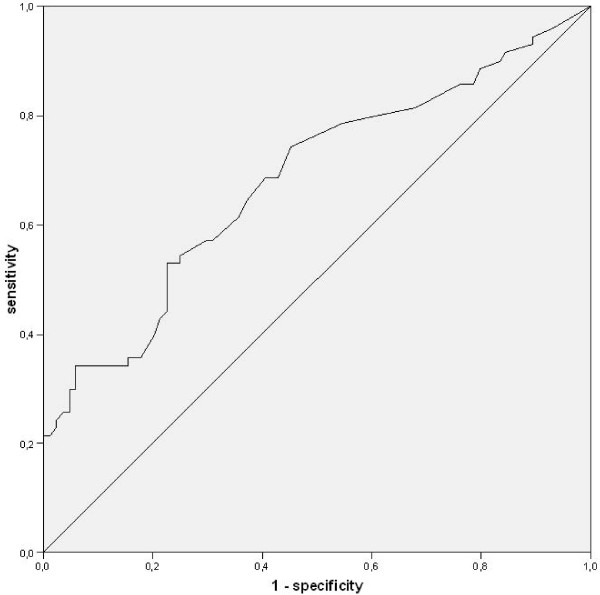
**ROC curve for measurement of FENO in the diagnosis of asthma (AUC = 0.645; 95%CI 0.559–0.731)**.

The highest FENO measures are attributed to the diagnosis asthma, as the box-plot illustrates (Figure 3). The area under the curve was 0.645 (95%CI 0.559–0.731; p = 0.002) if compared with a 20% fall of FEV1 after inhaling methacholine concentration ≤ 16 mg/mL and/or positive bronchodilator response. The results of the ROC analysis are illustrated in figure 4. The highest sum of sensitivity and specificity was given at cut-off 46 ppb (Table 2). The pre-test probability of 'having asthma' (46.9%) was enhanced up to a PPV of 80%. LR+ was highest at this cut-off point (Table 3). 30 (19.2%) patients had a FENO > 46 ppb. Therefore, five patients had to be diagnosed with the FENO analyser to save one bronchial provocation. There were no notable differences when patients with inhaled steroids were excluded from calculation, PPV remained 80%.

**Table 2:** Sensitivity (sens), specificity (spec), positive predictive value (PPV) and negative predictive value (NPV) at different cut-off points (n = 160); unit of FENO is parts per billion

**Asthma diagnoses**	**FENO**	**sens [%]** **(95%CI)**	**spec [%]** **(95%CI)**	**PPV [%]** **(95%CI)**	**NPV [%]** **(95%CI)**	**n**
Borderline BHR	> 12	85 (76–92)	24 (16–34)	50 (41–58)	65 (47–79)	126
mild BHR	> 20	64 (53–74)	58 (47–77)	57 (47–67)	65 (53–74)	82
moderate to severe BHR	> 35	32 (25–42)	84 (74–90)	63 (47–77)	58 (49–67)	38
positive bronchodilator reversibility	> 46	32 (23–43)	93 (85–97)	80 (63–91)	61 (52–69)	30
(n = 75)*	> 76	13 (7–23)	100 (96–100)	100 (72–100)	57 (49–65)	11

Mild BHR	> 12	90 (79–95)	25 (17–34)	40 (32–50)	81 (64–91)	126
moderate to severe BHR	> 20	67 (54–78)	62 (52–71)	50 (39–61)	77 (67–85)	82
positive bronchodilator reversibility	> 35	36 (25–49)	83 (75–89)	55 (40–70)	70 (61–77)	38
(n = 58)^§^	> 46	36 (25–49)	91 (84–95)	70 (52–83)	72 (63–79)	30
	> 76	17 (10–29)	100 (96–100)	100 (72–100)	68 (60–75)	11

*prevalence of asthma = 46.9%, prevalence of 'no asthma' = 53.1%

^§ ^prevalence of asthma = 36,3%, prevalence of 'no asthma' = 63.7%

**Table 3:** Likelihood ratio at different cut-off points (n = 160); unit of FENO is parts per billion; LR+ is positive likelihood ratio, LR- is negative likelihood ratio

**Asthma diagnoses**	**FENO**	**LR+ (95%CI)**	**LR-(95%CI)**
Borderline BHR, mild BHR, moderate to severe BHR, positive bronchodilator reversibility (n = 75)	> 12	1.12 (0.96–1.30)	0.62 (0.32–1.22)
	> 20	1.55 (1.12–2.14)	0.65 (0.47–0.91)
	> 35	1.94 (1.09–3.48)	0.81 (0.68–0.98)
	> 46	4.53 (1.96–10.49)	0.73 (0.62–0.86)
	> 76	not calculable	not calculable

Mild BHR, moderate to severe BHR, positive bronchodilator reversibility (n = 58)	> 12	1.19 (1.03–1.37)	0.42 (0.18–0.97)
	> 20	1.76 (1.30–2.39)	0.53 (0.36–0.79)
	> 35	2.17 (1.25–3.77)	0.77 (0.62–0.95)
	> 46	4.10 (2.02–8.36)	0.70 (0.57–0.86)
	> 76	not calculable	not calculable

At the cut-off point 76 ppb every patient would be correctly diagnosed as having asthma. 11 (6.9%) asthma patients had a FENO > 76 ppb. Therefore, nearly fifteen patients had to be diagnosed with the FENO analyser to save one bronchial provocation. It was not possible to exclude asthma (diagnosed with methacholine concentration ≤ 16 mg/mL) with this FENO cut-off value, as the pre-test probability of 'not having asthma' was within the 95% confidence interval of NPV. PPV was only 63% (95%CI 47–77) at the cut-off value > 35 ppb. At the cut-off point 12 ppb, which was established by Menzies et al. [18], we received very low PPV.

In a second step, we analyzed if FENO helped to identify asthmatics among all subjects with unsuspicious spirometric results (n = 101; not in table). 49 of these patients had asthma (pre-test probability 48.5%). The optimal cut-off with highest sum of sensitivity and specificity was at 46 ppb again. The sensitivity of FENO > 46 ppb for these patients with unsuspicious spirometry was 35% (95%CI 23–49%), specificity was 90% (95%CI 79–96%), PPV 77% (95%CI 47–90%) and NPV 59% (95%CI 49–70%). 24 (23.8%) patients had a FENO > 46 ppb. Therefore, four patients with unsuspicious spirometric results had to be diagnosed with the FENO analyser to save one bronchial provocation.

### Sensitivity analyses

Ten patients with COPD and three patients with overlap had already stopped smoking several years before the examination. Four of them had accumulated one to three pack years. The diagnoses of these four patients changed into asthma. The other patients had accumulated ten to 80 pack years (mean 37.2 ± 26.3) and were consequently labelled as COPD further on. If these diagnostic changes were taken into account, the ideal cut-off level would remain at 46 ppb. In this case, the sensitivity was 30% (95%CI 21–41%), specificity 93% (95%CI 85–97%), positive predictive value (PPV) 80% (95%CI 63–91), NPV 57% (95%CI 49–66%). At 83 ppb specificity was 100% (95%CI 96–100%) and PPV was 100% (95%CI 72–100) (not in table).

There was a slight change of the results when the data were analyzed solely with non-smokers (n = 110). Again, the ideal cut-off remained at 46 ppb with sensitivity 34% (95%CI 23–46%) and specificity 94% (95%CI 83–98%). PPV increased up to 88% (95%CI 69–96%) and NPV was 52% (95%CI 42–63%). At 65 ppb specificity was 100% (95%CI 93–100%) and PPV was 100% (95%CI 77–100%) (not in table). In patients reporting allergic rhinitis (n = 47), test accuracy of FENO increased slightly at FENO > 46 ppb. Sensitivity was 45% (95% 31–60), specificity 90% (95%CI 75–97), PPV 86% (95%CI 65–95), NPV 56% (95%CI 42–69).

If only patients with mild, moderate to severe BHR, and/or positive bronchodilator response were accepted for the diagnosis asthma, the best NPV was found at ENO ≤ 12 ppb. NPV was 81% (95% CI 64–91); and LR- was lowest at this cut-off point (Table 3). 34 patients had FENO ≤ 12 ppb. Therefore, five patients have to be diagnosed to save one bronchial provocation for excluding asthma. There was no improvement of test accuracy in patients with unsuspicious spirometric results (n = 101; not in table). The best cut-off was at 12 ppb again. Sensitivity was 89% (95%CI 75–96), specificity was 19% (95%CI 11–30), PPV was 39% (95%CI 29–50), NPV was 75 (95%CI 51–90) (not in table). 16 (15.8%) had a FENO ≤ 12 ppb. NPV increased up to 82% (95%CI 64–92) when patients with inhaled steroids were excluded from calculation.

## Discussion

To our knowledge, this is the first study evaluating the diagnostic accuracy of a portable FENO analyser based on an electrochemical sensor in a prospective design in primary care setting. At the highest sum of sensitivity and specificity, we found a reasonable cut-off point at > 46 ppb which allows diagnosing asthma with a PPV of 80%. At a cut-off point > 76 ppb, specificity and PPV was 100%, which means asthma can be ruled in with the highest certainty. Mild and moderate to severe asthma can be excluded with NPV 81%, when FENO ≤ 12 ppb.

Due to these findings, FENO measurement might have an impact on the diagnostic management of patients. Five patients have to be evaluated with FENO to save one bronchial provocation test for ruling in asthma, and five patients have to be evaluated to exclude mild and moderate to severe asthma. The number could be decreased for ruling in when used in patients with unsuspicious spirometric results. In that case four patients need to be evaluated with NioxMino^® ^to save one bronchial provocation. In patients with unsuspicious spirometric results (n = 101), 16 patients had FENO ≤ 12 ppb and 24 had FENO > 46 ppb. Therefore, altogether three patients have to be tested with FENO to save one bronchial provocation. In Germany, investigation by a pneumologist including bronchial provocation would costs around 110€ which need to be compared with the costs of three FENO measurements (102€; 34 € per measurement [38]). Thus it seems reasonable to perform spirometric investigation at first in patients suspected to suffer from asthma. FENO measurement could be performed if spirometry shows no signs of airway obstruction, in particular as bronchial provocation is time consuming, carries a small risk [7] and cannot be performed in general practices. Therapy with inhaled steroids should be initiated when FENO > 46 ppb due to the already demonstrated dose-response relationship [35,39]. Mild and moderate to severe asthma is excluded when FENO ≤ 12 ppb. Referral for bronchial provocation seems to be indicated for intermediate values when 12 ppb < FENO ≤ 46 ppb.

This dual strategy for primary care patients was already used in an observational study with 55 patients by Hewitt et al. [40]. In this study FENO cut-offs at 20 ppb and 35 ppb were used, which were established by reproducibility measurements with the Niox^® ^chemoluminescence analyser [24]. This is in contrast to our range of indifferent results from 12 to 46 ppb. Our results might be explainable in relation to a study by Alving et al. who evaluated the agreement of NioxMino^® ^and Niox^® ^[17]. They found the limits of 95% confidence interval of agreement were -9.8 and 8.0 ppb. They stated that from a clinical point of view, accuracy is more important in a FENO range close to a cut-off between healthy and disease (20–35 ppb). The sum of the upper limit of the 95% CI (8 ppb) and 35 ppb is close to our best cut-off point (46 ppb) to rule in asthma; and the difference of the 95%CI (-9.8 ppb) and 20 ppb is close to our best cut-off point (12 ppb) to rule out asthma. Therefore, our findings might be due to a discrete imprecision of the electrochemical sensor. However, also with a 20 ppb cut-off point exclusion of asthma would be possible, with lower NPV. Beside that, our results indicate that a more sure positive diagnosis of asthma might be provided with FENO at 46 ppb, as stated above. Further research with long term follow-up would be necessary to evaluate the effectiveness of the dual diagnostic strategy with different FENO cut-off points.

Our study has some limitations, the most important one being related to the conflicting evidence about the ideal cut-off of for bronchial provocation testing with methacholine, which might be due to variation in the populations studied and the severity of disease [30,31]. The lack of consensus is also reflected by the use of different cut-off values in different diagnostic studies [4,6,15,16], which is in contrast to the cut-off at 16 mg/mL as suggested by the ATS guideline [7]. We took this limitation into account by performing sensitivity analyses with exclusion of patients with borderline BHR. Despite the lack of an ideal 'gold standard', our finding implicates that it is possible in general practice to rule out mild and moderate to severe asthma with FENO measurement. Borderline BHR could not be excluded indeed.

It has been shown that the current clinical guideline recommended FEV_1_/VC cut-off at 0.70 might lead to substantial over-diagnosis of COPD [41]. However, most patients identified as COPD were heavy smokers (21 of 25 with at least ten pack years), and patients with asthma had positive bronchodilation response or positive bronchial provocation result. That makes a false diagnostics improbable from a clinical point of view. Another point of discussion is the correct classification of the eight patients with only incomplete bronchodilator response. The best way for differentiation would have been a long term follow-up with trials of inhaled steroids, which was not possible within the study design. Beside that, bronchial provocation in all patients might have been helpful for further differentiation. However, this was not allowed by the Ethics Committee due to the risk of severe bronchial spasm. Based on medical history and spirometry investigation, five patients were very similar to COPD (all were heavy smokers) and the remaining three were most probably asthma patients with fixed airway obstruction. The sensitivity analyses showed that the cut-off point of FENO remained the same when actually non-smoking patients initially labelled as COPD or overlap were classified as asthma patients with fixed airway obstruction. This might attenuate the potential limitation, in particular as this difficult diagnostic group was small.

We included all patients referred by the GPs, even patients with current high tobacco use. It has been shown that tobacco smoke decreases exhaled NO [34], which could lead to false negative diagnoses. However, the exclusion of smokers from our analyses showed similar results. This might be due to the lower tobacco use of the asthma patients in our sample, thus accompanied by lower rate of distorted results. Another distortion might be caused by inclusion of patients with nasal allergies (44.4%), which could lead to elevated FENO [36]. However, PPV increased in this group. Another critical point is the positive correlation of FENO with age and sex [42] which might lead to reduced diagnostic accuracy [43]. However, especially in general practice unselected patients appear with various complaints and various ages which does not allow pre-selection in using diagnostic devices. Therefore this can also be seen as strength of the present study since we tried to evaluate the diagnostic accuracy under clinical reality in primary care. A solution for enhancing diagnostic accuracy might be found when adjusted norm values could be established as was postulated by Taylor et al. [42]. Therefore, in the near future FENO might prove more useful in terms of accuracy and of cost-effectiveness in asthma sub-phenotypes, like allergic patients or cough variant asthma. Another limitation is due to the lower severity of disease which is typically found in primary care population. Most patients with asthma were identified with bronchial provocation. Thus, our results might not be applicable to subjects with more severe OAD as found in secondary or tertiary care. It was not possible to specify the alternate diagnosis of the patients with no OAD, which is also a typical problem of diagnostic studies in primary care. It was impossible to perform every investigation (e.g. gastroscopy to determine gastro-oesophageal reflux; x-ray) until a definite diagnosis could be made. This would not have been allowed by the Ethics Committee. However, this limitation does not alter the FENO results. A final limitation might be that we used only a single FENO measurement, whereas the mean of three measurements is recommended by the guidelines [23]. On the other hand, it was recently stated that one measurement is about as precise as three measurements [17]; and its also clinical reality that more than one measurement is too expensive for routine use in general practice.

## Conclusion

FENO measurement with a portable electrochemical analyzer seems to be effective for ruling in and ruling out asthma in general practice. Asthma could be ruled in satisfyingly with a cut-off at FENO > 46 ppb. Mild and moderate to severe asthma could be ruled out satisfyingly using FENO ≤ 12 ppb as a cut-off point. In sum, three patients with unsuspicious spirometric results have to be tested with FENO to save one bronchial provocation test. Therefore, FENO measurement might be reasonable as bronchial provocation is a time consuming procedure and carries a small risk of severe bronchospasm. Further research is necessary to evaluate the effectiveness of this dual diagnostic strategy. The number needed to diagnose might be improved when the diagnostic precision could be enhanced by future technical developments.

## Competing interests

The authors declare that they have no competing interests.

## Authors' contributions

AS designed the study, performed the analyses and wrote the manuscript. LG performed the FENO measurements and helped to write the manuscript. TS helped to interpret the data and to write the manuscript. LG helped with patient recruitment and writing. GL helped in statistics and writing. JS helped to write the manuscript. FJM made the final diagnoses as pneumologist and helped to write the manuscript.
