# 'Diagnosing Asthma in General Practice with Portable Exhaled Nitric Oxide Measurement – Results of a Prospective Diagnostic Study: FENO ≤ 16 ppb better than FENO ≤ 12 ppb to rule out mild and moderate to severe asthma

**DOI:** 10.1186/1465-9921-10-64

**Published:** 2009-07-07

**Authors:** Antonius Schneider, Lisa Tilemann, Tjard Schermer, Lena Gindner, Gunter Laux, Joachim Szecsenyi, Franz Joachim Meyer

**Affiliations:** 1 Department of General Practice and Health Services Research, University Hospital, University of Heidelberg, Heidelberg, Germany; 2 Department of Primary Care Medicine, Radboud University Nijmegen Medical Centre, Nijmegen, the Netherlands; 3 Department of Cardiology, Pulmonology and Angiology, Medical Centre, University of Heidelberg, Heidelberg, Germany

## Correction

In our study to evaluate the diagnostic accuracy of FENO measurement with NioxMino^® ^for the diagnosis of asthma in general practice, we found the cut-off at FENO ≤ 12 ppb to rule out mild and moderate to severe asthma with a negative predictive value of 81% (95%CI 64–91%) [1]. We oriented ourselves at the already established value of 12 ppb [2]. However, we overlooked in the ROC analysis that the overall diagnostic accuracy improves slightly when the cut-off is chosen at FENO ≤ 16 ppb (revised table two) [see table 1]. Negative likelihood ratio was 0.38 (95%CI 0.22–0.64) and positive likelihood ratio was 1.76 (95%CI 1.37–2.26) using the 16 ppb cut-off (revised table three) [see Table 2].

**Table 1:** Sensitivity (sens), specificity (spec), positive predictive value (PPV) and negative predictive value (NPV) at different cut-off points (n = 160); unit of FENO is parts per billion

**Asthma diagnoses**	**FENO**	**sens [%] (95%CI)**	**spec [%] (95%CI)**	**PPV [%] (95%CI)**	**NPV [%] (95%CI)**	**n**
Borderline BHR mild BHR moderate to severe BHR positive bronchodilator reversibility (n = 75)*	> 12	85 (76–92)	24 (16–34)	50 (41–58)	65 (47–79)	126
	> 16	69 (58–79)	53 (42–63)	57 (46–66)	66 (54–76)	92
	> 20	64 (53–74)	58 (47–77)	57 (47–67)	65 (53–74)	82
	> 35	32 (25–42)	84 (74–90)	63 (47–77)	58 (49–67)	38
	> 46	32 (23–43)	93 (85–97)	80 (63–91)	61 (52–69)	30
	> 76	13 (7–23)	100 (96–100)	100 (72–100)	57 (49–65)	11

Mild BHR moderate to severe BHR positive bronchodilator reversibility (n = 58)^§^	> 12	90 (79–95)	25 (17–34)	40 (32–49)	81 (64–91)	126
	> 16	79 (67–88)	55 (45–64)	50 (40–60)	82 (72–90)	92
	> 20	67 (54–78)	62 (52–71)	50 (39–61)	77 (67–85)	82
	> 35	36 (25–49)	83 (75–89)	55 (40–70)	70 (61–77)	38
	> 46	36 (25–49)	91 (84–95)	70 (52–83)	72 (63–79)	30
	> 76	17 (10–29)	100 (96–100)	100 (72–100)	68 (60–75)	11

*prevalence of asthma = 46.9%, prevalence of 'no asthma' = 53.1%

^§ ^prevalence of asthma = 36,3%, prevalence of 'no asthma' = 63.7%

**Table 2:** Likelihood ratio at different cut-off points (n = 160); unit of FENO is parts per billion; LR+ is positive likelihood ratio, LR- is negative likelihood ratio

**Asthma diagnoses**	**FENO**	**LR+ (95%CI)**	**LR- (95%CI)**
Borderline BHR, mild BHR, moderate to severe BHR, positive bronchodilator reversibility (n = 75)	> 12	1.12 (0.96–1.30)	0.62 (0.32–1.21)
	> 16	1.47 (1.12–1.93)	0.58 (0.39–0.86)
	> 20	1.55 (1.12–2.14)	0.65 (0.47–0.91)
	> 35	1.94 (1.09–3.48)	0.81 (0.68–0.98)
	> 46	4.53 (1.96–10.49)	0.73 (0.62–0.86)
	> 76	not calculable	not calculable

Mild BHR, moderate to severe BHR, positive bronchodilator reversibility (n = 58)	> 12	1.19 (1.03–1.37)	0.42 (0.18–0.97)
	> 16	1.76 (1.37–2.26)	0.38 (0.22–0.64)
	> 20	1.76 (1.30–2.39)	0.53 (0.36–0.79)
	> 35	2.17 (1.25–3.77)	0.77 (0.62–0.95)
	> 46	4.10 (2.02–8.36)	0.70 (0.57–0.86)
	> 76	not calculable	not calculable

In patients with unsuspicious spirometric results (n = 101; not in table) there was no improvement of diagnostic accuracy. The best cut-off point was at FENO ≤ 16 ppb again. In this diagnostic group sensitivity was 78% (95%CI 63–89%), specificity was 45% (95%CI 34–57%), PPV was 45% (95%CI 34–57%) and NPV was 78% (95%CI 63–89%).

Table two [see Table 1 below] illustrates that the patient group with correctly excluded asthma by FENO measurement increases at FENO ≤ 16 ppb; and the range of the confidence interval narrows. Thus three patients need to be diagnosed for excluding asthma in order to save one bronchial provocation test when FENO ≤ 16 ppb is used as the cut-off point. With FENO ≤ 12 ppb five patients need to be tested in order to exclude asthma in one of them. Therefore, we suggest choosing FENO ≤ 16 ppb to rule out mild and moderate to severe asthma. This improves diagnostic efficiency compared to the ≤ 12 ppb cut-off point.

We would like to correct the following points in the manuscript:

In the **Results **section of the **Abstract **lines 6–7 should read as:

"16 ppb (n = 68; 42.5%), sensitivity was 79% (95%CI 67–88), specificity 55% (95%CI 45–64), PPV 50% (95%CI 40–60), NPV 82% (95%CI 72–90)".

Also in line 7, "Three" should say "Two".

In the **Conclusion **section of the **Abstract**, in line 2, "FENO ≤ 12 ppb" should say "FENO ≤ 16 ppb".

In the **Sensitivity analyses **section, in line 2 of the third paragraph, "FENO ≤ 12 ppb" should say "FENO ≤ 16 ppb", "81% (95% CI 64–91)" should say "82% (95% CI 72–90)" and "34" should say "68". In line 3, "FENO ≤ 12 ppb" should say "FENO ≤ 16 ppb" and "five" should say "three". In line 4 "12 ppb" should say "16 ppb". The sentence starting in line 5 and ending in line 6 should read: "Sensitivity was 78% (95%CI 63–89), specificity was 45% (95%CI 34–57), PPV was 45% (95%CI 34–57), NPV was 78 (95%CI 63–89)". In line 6, "16 (15.8%)" should say "37 (36.6%)", "FENO ≤ 12 ppb" should say "FENO ≤ 16 ppb" and "increased up to 82% (95%CI 64–92)" should say "was 77% (95%CI 61–88)".

In the **Discussion **section, in line 4, "81%" should say "82%" and in line 5, "FENO ≤ 12" should say "FENO ≤ 16"

In the second paragraph, in line 1, "five" should say "three". In line 5, "16 patients had FENO ≤ 12 ppb" should say "37 patients had FENO ≤ 16 ppb". Also in line 5, "three" should say "two" and in lines 11 and 12 "FENO ≤ 12 ppb" should say ""FENO ≤ 16 ppb" and 12 ppb<FENO should say 16 ppb<FENO.

In the third line of the third paragraph "12 to 46 ppb" should say "16 to 46 ppb" and in the seventh line, the second half of the sentence that reads **"**and the difference of the 95%CI (-9.8 ppb) and 20 ppb is close to our best cut-off point (12 ppb) to rule out asthma" should not be there.

In the **conclusion **section, in line 3 "FENO ≤ 12 ppb" should say ""FENO ≤ 16 ppb" and "three" should say "two".
